# Effects of Vitamin D3 Supplementation and Aerobic Training on Autophagy Signaling Proteins in a Rat Model Type 2 Diabetes Induced by High-Fat Diet and Streptozotocin

**DOI:** 10.3390/nu15184024

**Published:** 2023-09-17

**Authors:** Hadi Golpasandi, Mohammad Rahman Rahimi, Slahadin Ahmadi, Beata Łubkowska, Paweł Cięszczyk

**Affiliations:** 1 Department of Exercise Physiology, University of Kurdistan, Sanandaj 66177-15175, Iran; hadi.golpasandi@uok.ac.ir; 2 Department of Physiology and Pharmacology, School of Medicine, Kurdistan University of Medical Sciences, Sanandaj 66186-34683, Iran; slahadin@gmail.com; 3 Faculty of Health and Life Sciences, Gdansk University of Physical Education and Sport, Gorskiego 1, 80-336 Gdansk, Poland; beata.lubkowska@awf.gda.pl (B.Ł.); pawel.cieszczyk@awf.gda.pl (P.C.)

**Keywords:** aerobic exercise training, excessive autophagy, Type 2 diabetes, vitamin D3 receptor, high fat diet

## Abstract

The aim of this study was to investigate the combined effects of vitamin D3 supplementation and aerobic training on regulating the autophagy process in rats with type 2 diabetic induced by a high-fat diet and streptozotocin. A total of 40 Wistar rats were divided into five groups: normal control (NC), diabetic control (DC), diabetic + aerobic training (DAT), diabetic + vitamin D3 (DVD), and diabetic + aerobic training + vitamin D3 (DVDAT). The rats underwent eight weeks of aerobic training with an intensity of 60% maximum running speed for one hour, along with weekly subcutaneous injections of 10,000 units of vitamin D3. The protein levels of different autophagy markers were assessed in the left ventricular heart tissue. The results showed that the protein levels of AMPK, pAMPK, mTOR, and pmTOR were significantly lower in the DC group compared to the NC group. Conversely, the levels of ULK, Beclin-1, LC3II, Fyco, and Cathepsin D proteins were significantly higher in the DC group. However, the interventions of aerobic training and vitamin D3 supplementation, either individually or in combination, led to increased levels of AMPK, pAMPK, mTOR, and pmTOR, and decreased levels of ULK, Beclin-1, LC3II, Fyco, and Cathepsin D (*p* < 0.05). Additionally, the aerobic capacity in the DAT and DVDAT groups was significantly higher compared to the NC, DC, and DVD groups (*p* < 0.05). These findings suggest that type 2 diabetes is associated with excessive autophagy in the left ventricle. However, after eight weeks of vitamin D3 supplementation and aerobic training, a significant reduction in excessive autophagy was observed in rats with type 2 diabetes.

## 1. Introduction

Diabetes, a highly prevalent metabolic disorder, can adversely affect heart tissue and contribute to the development of diabetic cardiomyopathy (DCM). DCM is a leading cause of mortality in individuals with type 2 diabetes (T2D) [1,2]. DCM was first described as a distinct disease in individuals with diabetes who had symptoms of heart failure in 1972 [3]. In DCM, there is insulin resistance in heart tissue, hyperinsulinemia, and the development of hyperglycemia, which can lead to apoptosis in cardiomyocytes [4]. Autophagy is a process in cells that breaks down and removes old proteins and parts. There are three different types of autophagy: macro-autophagy, micro-autophagy, and chaperone-mediated autophagy and it plays an important role in preserving cardiomyocytes [5]. However, hyperglycemia and dyslipidemia can influence the autophagy process in cardiomyocytes through the AMPK and mTOR signaling pathways [6]. Dyslipidemia decreases “macro-autophagy/autophagy” in cardiomyocytes via inhibiting the cellular energy-sensing AMPK and stimulation of mTOR signaling [7]. This event can occur through the inhibition of ULK-1, one of the initiating factors of autophagy [6]. AMPK activates autophagy by inhibiting mTOR and stimulating ULK-1 [6]. The activation of ULK-1, in turn, leads to the phosphorylation of the Beclin-1-PI3k type III complex, the formation of the phagophore, and the nucleation process [8,9]. After the nucleation stage, the process of autophagy and its elongation occurs through the LC3I and LC3II complex, resulting in the formation of an autophagosome [10]. Kanamori et al. demonstrated autophagic flux by increasing the accumulation of LC3-II and simultaneously reducing the number of autolysosomes and lysosomes in myocardial cells affected by T2D, such that the increase in LC3-II levels was accompanied by the suppression of autophagy in the final stage of the autophagy process [11]. Another study also showed a weak autophagic response because of the reduction in LC3II levels in the cardiomyocytes of obese rats induced by high fat diet (HFD) [12]. Nevertheless, other studies have claimed that insulin resistance could cause autophagy to be more active through positive regulation of LC3II and beclin1, which could ultimately endanger the survival of cardiac cells in T2D [13,14]. Therefore, it seems that autophagy plays a dual role in T2DM, as both suppression and overactivity of autophagy can have a pathological effect on cardiac cells in diabetes. Hence, the precise role of autophagy in the heart of type 2 diabetes is still unclear.

The role of physical activity in improving physical performance, general health, and the prevention and treatment of certain pathological conditions has been demonstrated [15]. The occurrence of autophagy is prompted by stress factors, like starvation or physical activity. Autophagy helps to fix old or broken proteins and parts of cells. It also helps to create new parts of cells and a type of energy called ATP. For example, one of the main mechanisms of cellular adaptation to stress is positive or negative regulation of autophagy. When cellular stress increases during training, autophagy increases to provide energy substrates and adapt cellular structures to new demands [16].

In the heart, normal levels of autophagy act as a homeostatic mechanism that maintains cardiac structure and function [17]. However, the effect of aerobic training on the expression of autophagy markers in T2D has not been sufficiently investigated [18,19]. Previous findings have shown that 8 weeks of intense training can enhance autophagy by increasing the expression of beclin1, ULK1, and LC3 proteins in the left ventricular muscle tissue of rats with T2D [18]. One study reported an increase in autophagosome production through an increase in LC3II protein content in rats with obesity induced by a HFD combined with exercise training compared to the obese control group [19].

In addition to exercise training, nutritional supplements can also be beneficial in improving diabetic conditions [20]. In animal studies, vitamin D3 deficiency is associated with cardiac remodeling activities such as altered energy metabolism, cardiac inflammation, oxidative stress, fibrosis and apoptosis, cardiac hypertrophy, left ventricular alterations, and contractile dysfunction [21]. However, the effects of Vit D3 on left ventricle and related cellular mechanisms are inadequately defined. 

A growing number of studies have shown that vit D3 preserves cell survival, reduces cell apoptosis, and decreases disease severity through autophagy regulation [22]. Yao et al. demonstrated that vit D3 inhibits cell death caused by impaired autophagy function and improves cardiac function [23]. Ho et al. (2016) also showed in their study that vit D3 treatment improves autophagy by reducing the expression of Beclin-1, LC3-II, and p62 proteins in the myocardium of rats treated with vit D3 [22]. They also demonstrated that vit D3 can improve heart function by inhibiting cardiac inflammatory infiltration, reducing apoptosis of myocardial cells, and regulating autophagy. Considering the importance of vit D3 in glucose homeostasis and autophagy regulation, no study has yet evaluated the potential effect of its combination with regular aerobic training on the autophagy process in the cardiac tissue of rats with Type 2 diabetes. Therefore, given the high prevalence of vit D3 deficiency in diabetes and to clarify their relationship, our assumption is that implementing a period of aerobic training simultaneously with vit D3 supplementation is likely to regulate autophagy-related factors in rats with Type 2 Diabetes. The aim of this research is to examine the impact of vitamin D3 supplementation and aerobic training on autophagy signaling proteins in a rat model of type 2 diabetes induced by a high-fat diet and streptozotocin.

## 2. Materials and Methods

### 2.1. Preparation of Laboratory Animals and Their Maintenance

The present study was an experimental study with a post-test control group design. Forty male Wistar rats with a mean age of 6 weeks and weight of 200–250 g were obtained from the animal research center of the Pasteur Institute of Iran after obtaining approval from the committee on ethics and research in laboratory animals (Code: IR.UOK.REC.1400.015). All experimental procedures were performed under the NIH guidelines (2020) for the care and use of laboratory animals [24]. Male Wistar rats were placed in groups of four in the animal house under controlled temperature (22 ± 2 °C) in transparent polycarbonate cages and subjected to a 12-h light/dark cycle with free access to drinking water. After the acclimation period, the rats were randomly divided into two groups, i.e., normal control and diabetic groups.

### 2.2. Induction of Diabetes Cardiomyopathy in Rat Model and Grouping Based on This

Based on previous studies, the model applied in the present study to establish T2DM in male Wistar rats via the combination of HFD+ STZ was used [25,26,27]. In this regard, the rats received a HFD consisting of fat (45%), carbohydrates (35%), and protein (20%) for 6 weeks [25], followed by an intraperitoneal (IP) (i.p.) injection of a freshly prepared solution of STZ in citrate buffer at pH 4.5 [25]. Diabetic rats continued to receive the high-fat diet until the end of the protocol. To evaluate the diabetic status of the rats, their fasting blood glucose levels were measured 72 h after STZ injection [25]. Rats with blood glucose levels >250 mg/dl were considered diabetic [28]. Serum glucose levels in the control group remained within the normal range (80–100 mg/dL) throughout the study period. Rats with high blood sugar levels over 250 mg/dL were seen as having diabetes. The levels of sugar in the blood of the control group stayed normal (between 80 and 100 mg/dL) during the whole study. Afterwards, the rats with diabetes were split into 4 groups in a random way: diabetic control (DC), diabetic + aerobic training (DAT), diabetic + vit D3 (DVD), and diabetic + aerobic training + vit D3 (DVDAT).

### 2.3. Measuring Maximum Running Speed and the Aerobic Training Protocol

After two weeks of familiarization, rats in the training groups performed a graded exercise test on a treadmill (TR105, Maze Router, Urmia, IRAN) to measure their maximum running speed (MRS) and time to exhaustion (TTE). The standard incremental treadmill test by Bedford et al. (1979) was used to measure maximum running speed [29]. The test consists of 10 stages of 3-min durations each, starting at a speed of 3.0 km/h with zero incline; then, speed was increased 0.3 km/h in each stage until the point of exhaustion. The speed obtained in the last completed stage was considered as the rat’s maximum running speed equivalent to maximal oxygen consumption (Vo_2max_). The test was performed once every two weeks.

After estimating the maximum running speed of the rats, the training groups ran on a treadmill for 8 weeks, 5 sessions per week, each session lasting 30–60 min with intensity of 55–65% of the maximum speed obtained through the MRS test (Table 1) [30].

### 2.4. Vitamin D3 Supplementation Protocol

**Figure 1:**
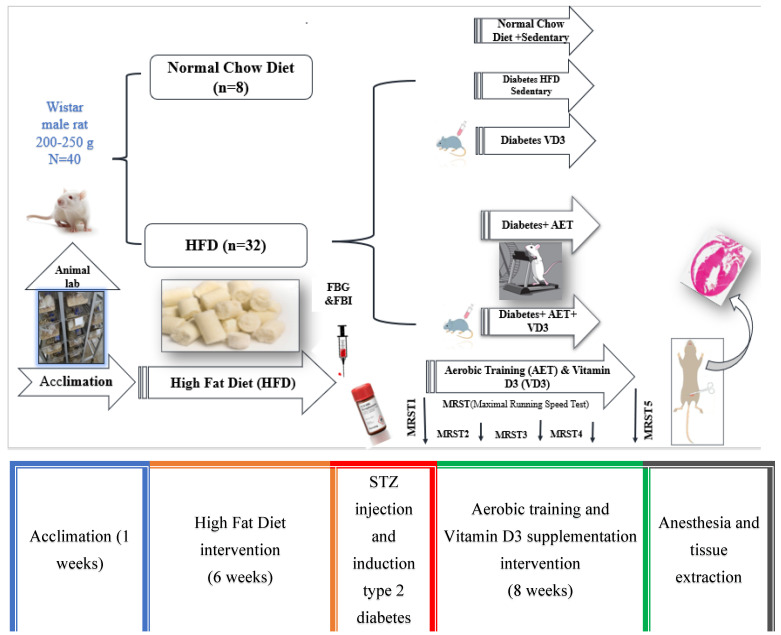
A summary panel to describe the study design. The first part of the experiment was designed to determine the acclimation and high-fat diet (HFD) (6 weeks), and the second part was designed to perform 8 weeks of aerobic training (AET), vit D3 supplementation (VD3) and maximal running speed test (MRST) in rats.

The rats were divided into two groups, DVD and DVDAT, and received vit D3 dissolved in sesame oil at a dose of 10,000 (IU/kg) (Caspian Tamin Company, Rasht, Iran, Batch Number: 699) subcutaneously once a week [31]. The experimental design of the present study is shown in Figure 1.

### 2.5. Western Blot

Seventy-two hours after the last training session and after an overnight fast of 8 h, all rats were anesthetized using a combination of ketamine (75 mg/kg) and Xylazine (10 mg/kg). We measured protein expression in heart extracts using western blot analysis. The left ventricle of the heart was homogenized in liquid nitrogen, and protein extraction was performed using lysis buffer. The samples were then separated and centrifuged. The resulting protein liquid was clear and stored at −20 °C. To determine the protein content, we used the Bradford method. The proteins were separated using a specialized gel and transferred to another material. The membrane was blocked with 5% BSA in TBST to prevent non-specific bindings. Finally, the membrane was incubated overnight at 4 °C with primary antibodies, including AMPKα1/2 (SANTA CRUZ BIOTECHNOLOGY, sc-74461), phospho-AMPKα1/2 (Thr 172) (SANTA CRUZ BIOTECHNOLOGY, sc-33524), BECN1 or Beclin-1 (E-8) (SANTA CRUZ BIOTECHNOLOGY, sc-48341), cathepsin D (C-5) (SANTA CRUZ BIOTECHNOLOGY, sc-377124), FYCO1 (Novus Biologicals, NBP1-47266), LC3B (Cell Signaling Technology, 2775), mTOR (30) (SANTA CRUZ BIOTECHNOLOGY, sc-517464), phospho-mTOR (59. Ser 2448) (SANTA CRUZ BIOTECHNOLOGY, sc-293133), ULK1 (F-4) (SANTA CRUZ BIOTECHNOLOGY, sc-390904) (Biotechnology company, Dallas, Texas, USA), and VDR Polyclonal (E-AB) (Elabscience,60676) (Elabscience company, Houston, Texas, USA), purchased from Cell Signaling Technology (1:500). Afterward, the membranes were subjected to three rounds of cleaning and then immersed in a mixture of secondary antibodies and 5% milk in TBST at room temperature for one hour. We utilized specific chemicals and films to visualize the protein bands. The protein amount was quantified using a computer program called Image J. In simpler terms, after undergoing electrophoresis on three separate gels and staining with a blue dye, each lane contained the appropriate protein based on its weight. The gel pieces were then placed in small tubes and soaked in 200 mL of wash buffer. They were kept at 30 °C and shaken in a machine overnight to develop. Afterward, the tubes were spun at high speed for 10 min, and the liquid portion was transferred to small tubes commonly used in modern laboratories. These liquids were tested using a specialized machine called SDS-PAGE electrophoresis along with Western blotting.

### 2.6. Statistical Analysis

We used GraphPad Prism version 9 software, made by GraphPad Software in San Diego, USA, to analyze the data. The mean percentage changes were calculated using this formula. Results of one-way analysis of variance (ANOVA) with repeated measures was used to compare changes in rats’ performance, and one-way ANOVA was used to assess changes in protein levels involved in autophagy. Additionally, a post hoc Bonferroni test was used to evaluate pairwise differences between groups. All data were presented as mean± SEM, PMC and displayed in bar graphs. Statistical significance was considered at *p* < 0.05.

Mean Percentage Changes = (new_value − original_value)/|original_value| × 100

## 3. Results

### 3.1. Effects of Aerobic Training in Combination with Vitamin D3 Supplementation on Time-to Exhaustion (TTE)

**Figure 2:**
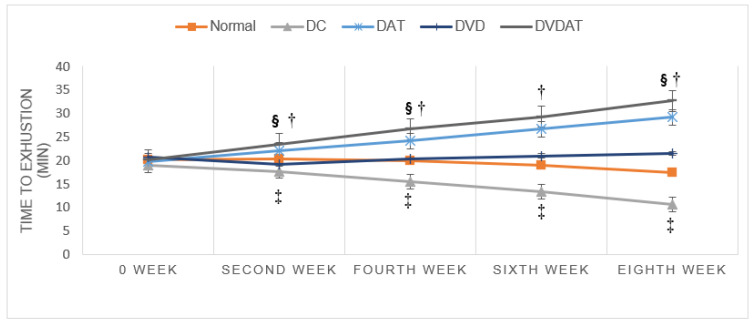
Results related to Time to Exhaustion (TTE) during the different periods of AT and combination AT and VitD3 in T2D rats. *p* < 0.05, (§) significant compared to the control diabetes group, (†) significant compared to the T2D + VD3, ‡ significant compared to normal control.

Results of ANOVA with repeated measures showed significant effects of time (F = 28.155, *p* < 0.000, ƞ^2^ = 0.53), group (F = 30.983, *p* < 0.000, ƞ^2^ = 0.83), and time-group interaction (F = 73.240, *p* < 0.000, ƞ^2^ = 0.92) on TTE, with a 49.57% increase in the DAT group, a 62% increase in the DVDAT group, and a decrease 43.8% in the DC group compared to baseline (pre-test). The results indicated improved physical performance in the DAT and DVDAT groups compared to the DC group, but no significant difference was observed between the DAT and DVDAT groups (*p* > 0.076) (Figure 2).

### 3.2. Effects of Aerobic Training along with Vitamin D3 Supplementation on p-AMPK, p-AMPK/AMPK, mTOR, and pm-TOR Proteins

**Figure 3:**
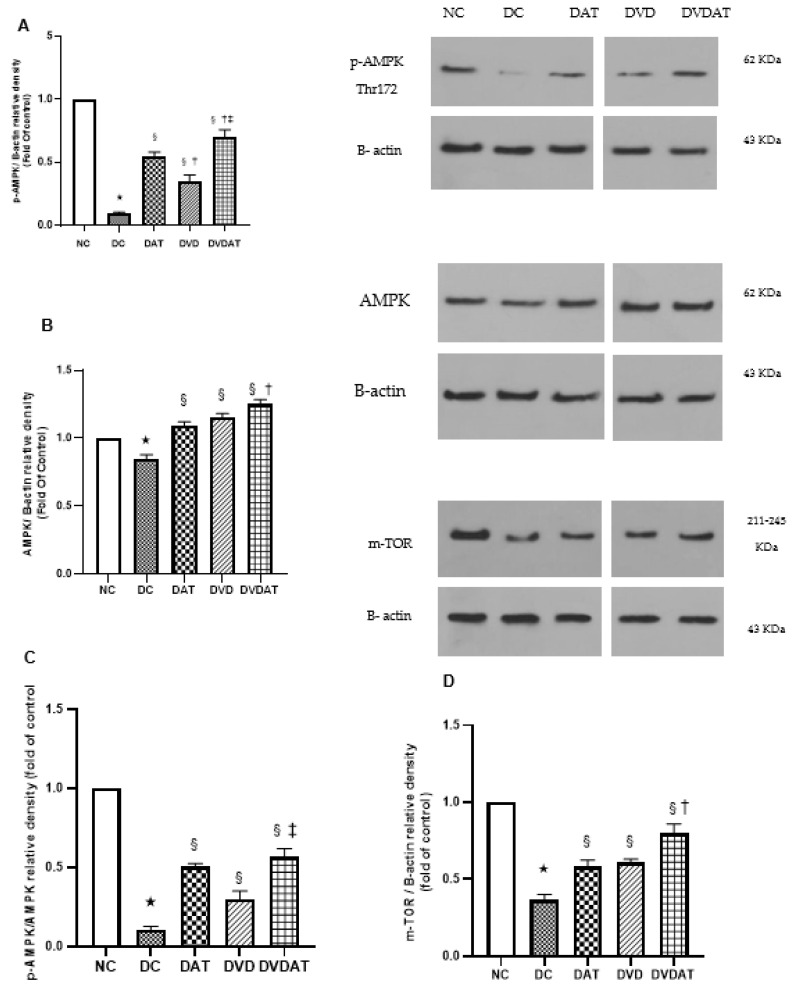

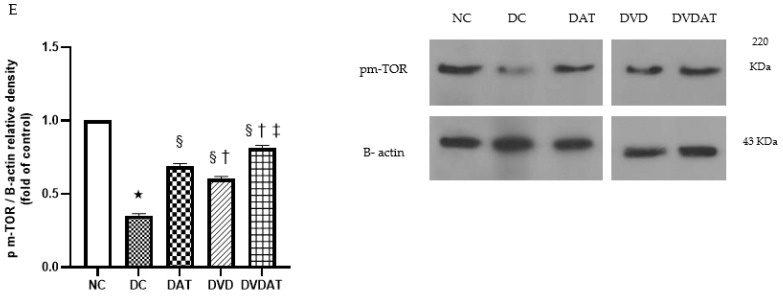
The effect of aerobic training and Vitamin D3 supplementation on protein expression involved in autophagy regulator in left ventricle. Quantification graphs of (**A**) p-AMPK, (**B**) AMPK, (**C**) pAMPK/AMPK ratio, (**D**) m-TOR and (**E**) pmTOR proteins relative to B-actin. Data are represented as means ± SEM (n = 8), *p* < 0.05; significant difference between groups indicated by (*): Significantly compared to the Normal diet group, (§): Significantly compared to the Diabetes control group, (†): Significantly compared to the aerobic training group and (‡) Significantly compared to the Vitamin D3; NC: Normal Control (n = 8); DC: Diabetic Control (n = 8); DAT: Diabetic + Aerobic Training (n = 8); DVD: Diabetic + VitaminD3 (n = 8); DVDAT: Diabetic + VitaminD3 + Aerobic training (n = 8).

The regulatory factors of autophagy, including pAMPK, pAMPK:AMPK ratio, mTOR, and pmTOR, were investigated. In the DC group, a significant decrease of 91% in pAMPK protein level, 90% in pAMPK:AMPK ratio, 64% in mTOR, and 65% in pmTOR proteins level compared to the NC group was observed (*p* < 0.000) (Figure 3A–D). Interventions with DAT, DVD, and DVDAT resulted in a significant increase in the pAMPK protein content (511.1%, 277.7%, and 762.5%, respectively) and in the pAMPK:AMPK ratio (400%, 190% and 460%, respectively) compared to the DC group (*p* < 0.000) (Figure 3A,B). The results related to mTOR and pmTOR protein content indicated significant differences among the present research groups (F = 29.14, F = 130.5, *p* < 0.000, ƞ^2^ = 0.95), with significant increases observed in the DAT group (61.1% and 94.2%), DVD group (66.6% and 71.43%), and DVDAT group (119.4% and 125.7%, respectively) compared to the DC group (Figure 3C,D).

### 3.3. Effects of Aerobic Training along with Vitamin D3 Supplementation on ULK-1, Beclin-1, LC3II, Fyco-1 and Cathepsin D protein Levels

**Figure 4:**
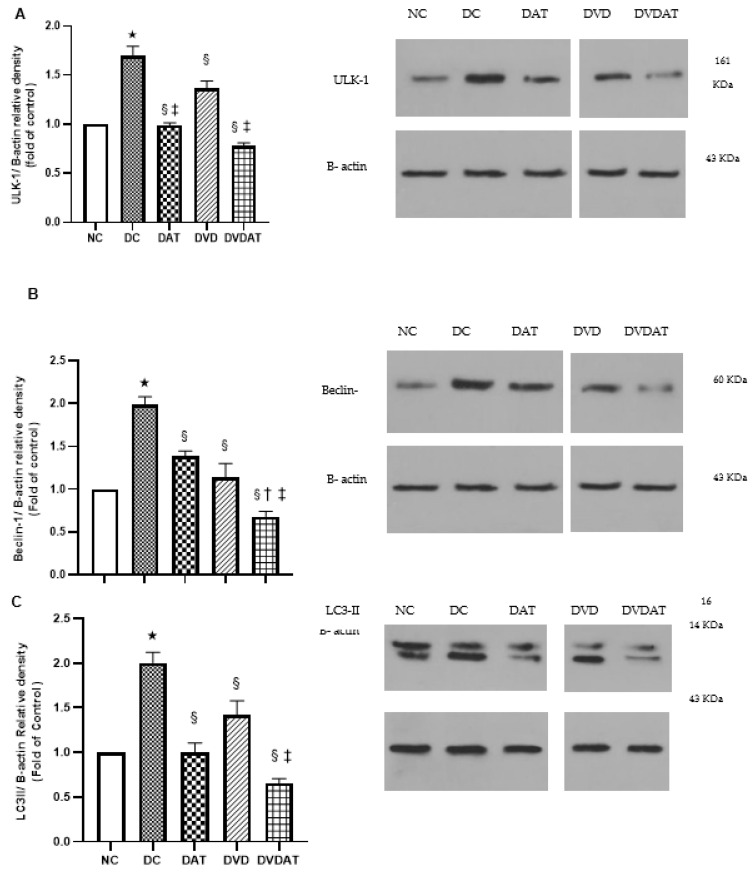

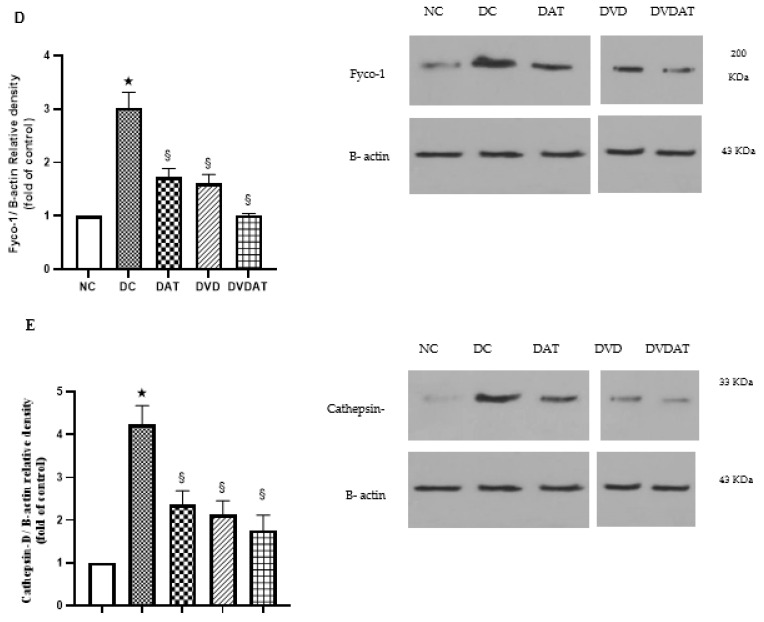
The effect of aerobic training and Vitamin D3 supplementation on protein expression involved in autophagy in left ventricle. Quantification graphs of (**A**) ULK-1, (**B**) Beclin-1, (**C**) LC3II, (**D**) Fyco-1 and (**E**) Cathepsin D proteins relative to B-actin. Data are represented as means ± SEM, (n = 8) and significant difference between groups indicated by (*): Significantly compared to the Normal diet group, (§): Significantly compared to the Diabetes control group, (†): Significantly compared to the aerobic training group and (‡) Significantly compared to the Vitamin D3; NC: Normal control (n = 8); DC: Diabetic Control (n = 8); DAT: Diabetic + Aerobic Training (n = 8); DVD: Diabetic VitD3 (n = 8); DVDAT: Diabetic + VitD3 + Aerobic training (n = 8).

To evaluate the markers involved in autophagy, the protein contents of ULK-1, Beclin-1, LC3-II, Fyco-1, and Cathepsin-D were assessed. The results showed that in the DC group, the protein contents of autophagy markers increased significantly (69%, 98%, 100%, 202%, and 322%, respectively) compared to the NC group (*p* < 0.000) (Figure 4A–E). In addition, the DAT group showed significant decreases in ULK-1 (42%), Beclin-1 (−29.8%), LC3-II (−51.2%), Fyco-1 (−44.3%), and Cathepsin-D (−44.3%) compared to the DC group (Figure 4A–E). In the DVD group, there were decreases in ULK-1 (−20.7%), Beclin-1 (30.3%), LC3-II (50%), Fyco-1 (43%), and Cathepsin-D (44%), and in the DVDAT group, decreases were observed in ULK-1 (54.4%), Beclin-1 (66.1%), LC3-II (67.5%), Fyco-1 (66.5%), and Cathepsin-D (58.2%) compared to the DC group (*p* < 0.001), with greater reductions observed in ULK-1, Beclin-1, and LC3-II in the DVDAT group compared to the DAT and DVD groups (Figure 4A–E).

### 3.4. Effects of Aerobic Training along with Vitamin D3 Supplementation on VDR Protein

**Figure 5:**
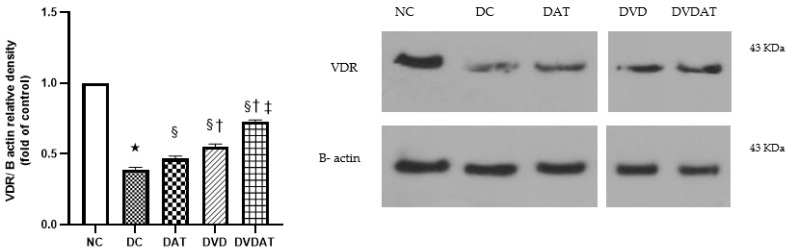
The effect of aerobic training and Vitamin D3 supplementation on Vitamin D3 receptor protein expression (VDR) in left ventricle. Quantification graphs of VDR proteins relative to B-actin. Data are represented as means ± SEM (n = 8) and significant difference between groups indicated by (*): Significantly compared to the Normal diet group, (§): Significantly compared to the Diabetes control group, (†): Significantly compared to the aerobic training group and (‡) Significantly compared to the Vitamin D3; NC: Normal control (n = 8); DC: Diabetic Control (n = 8); DAT: Diabetic + Aerobic Training (n = 8); DVD: Diabetic VitD3 (n = 8); DVDAT: Diabetic + VitD3 + Aerobic training (n = 8).

The results related to the evaluation of vitamin D3 receptors showed significant differences in the protein content of VDR among the study groups (F = 204.5, *p* < 0.000, ƞ^2^ = 0.97). In the DC group, a 61% decrease in VDR protein content was observed compared to the NC group (*p* < 0.000). However, in the DAT, DVD, and DVDAT groups, a significant increase in VDR protein content was observed (*p* < 0.05), with the greatest increase observed in the DVDAT group compared to the DAT and DVD groups (56.5% and 30.9%) (*p* < 0.000) (Figure 5).

## 4. Discussion

### 4.1. Impact of Type 2 Diabetes Mellitus on Left Ventricular Heart Tissue

The present study is the first to investigate the effects of 8 weeks of aerobic training in combination with vitamin D3 supplementation on autophagy regulatory markers in the left ventricle tissue of streptozotocin-induced diabetic cardiomyopathy in rats. The results showed a significant reduction in p-AMPK and the ratio of p-AMPK to AMPK in the DC group compared to the CO group, which is consistent with the findings of other studies [32,33,34,35]. AMPK becomes active during cellular stress because of an increase in the AMP-to-ATP ratio, thus regulating cellular energy homeostasis [36]. AMPK promotes autophagy by direct phosphorylation of autophagy-related proteins and indirectly via regulating the expression of autophagy-related genes [34].

Based on the findings mentioned, it seems that diabetic cardiomyopathy in the present study may have resulted in excessive autophagy activity, characterized by increased levels of autophagy markers and decreased protein contents of p-AMPK and p-AMPK/AMPK ratio in the DC group compared to the NC group. One of the mechanisms involved in the decreased p-AMPK in the DC group may be the inhibition of AMPK activity by increased levels of ULK-1 through a negative feedback mechanism. Moreover, hyperglycemia can lead to cardiac dysfunction and diabetic cardiomyopathy through oxidative stress and inflammation. It has been shown that reduced AMPK activity is associated with impaired glucose uptake and increased oxidative stress in the heart, both of which contribute to the progression of diabetic cardiomyopathy. Therefore, the observed decrease in AMPK level in the DC group of rats may be caused by excessive autophagy in response to high levels of oxidative stress and inflammation associated with diabetes. However, the lack of investigation into oxidative stress and inflammation markers may be one of the limitations of the present study.

Furthermore, a significant increase in the protein levels of ULK-1, Beclin-1, LC3-II, Fyco-1, and Cathepsin-D was observed in the DC group compared to the NC group, which is consistent with the findings of Jafari et al. [18] and Jokar et al. [37].

Beclin-1, as a class III phosphatidylinositol 3-kinase complex, plays a crucial role in the nucleation and formation of phagophore in autophagy [38], which can be activated upstream by the ULK-1 complex or directly by AMPK [39]. However, the significant increase of Beclin-1 in the diabetes group in the present study indicates AMPK-independent autophagy activation. Overactive autophagy can lead to increased severity of pathological disorders in various diseases, such as Alzheimer’s [40], diabetic cardiomyopathy [41], and cardiovascular diseases [42]. In the current study, excessive autophagy causes increased protein levels of ULK1, Beclin1, LC3II, Cathepsin D, and Fyco1 in diabetic cardiomyopathy, indicating the important role of these proteins in different stages of the autophagy process.

ULK1 and Beclin1 are crucial for the initiation of autophagy, while LC3II plays a role in the formation of autophagosomes, which are cellular structures that engulf and degrade cellular components [43,44]. Cathepsin D is a lysosomal enzyme that degrades the contents of autophagosomes, and Fyco1 plays a role in the transport of autophagosomes to lysosomes for degradation [45,46]. Christian Kuhn et al. demonstrated in their study that increased expression of Fyco1 induces autophagy in rat cardiomyocytes, thereby protecting heart cells in response to mechanical stress caused by heart failure [47].

### 4.2. Effects of Aerobic Training on the Levels of Autophagy-Related Proteins in Rats with Diabetic Cardiomyopathy

Regarding the effects of 8 weeks of aerobic training on pAMPK levels, the findings indicated a significant increase of 511.1% in the DAT group and 762.5% in the DVDAT group compared to the DC group. Furthermore, a significant increase of 400% and 460% in the pAMPK/AMPK ratio was observed in the DAT and DVDAT groups, respectively, which is consistent with the results of Diniz et al. [48], Jin et al. [49], Asokan et al. [50], and de Bem et al. [51], but inconsistent with the findings of Choi et al. [52]. It has recently been shown that exercise training activates cellular signaling pathways associated with autophagy, including AMPK phosphorylation, which regulates cellular homeostasis and eliminates detrimental factors in cells [53].

Recent findings have also emphasized the role of AMPK in stimulating cardiac autophagy by increasing the levels of autophagy-related proteins [54,55]. Interestingly, in the present study, we demonstrated a significant increase in the protein content of p-AMPK and the pAMPK/AMPK ratio in response to aerobic training and aerobic training in combination with VD3, which was accompanied by a decrease in the levels of autophagy-related proteins. Therefore, it appears that the increase in p-AMPK resulting from aerobic training had a different effect on autophagy regulation in diabetic cardiomyopathy in the present study.

Increased levels of reactive oxygen species (ROS) through mitochondrial DNA damage and lipid peroxidation contribute to the development of diabetic cardiomyopathy [56], which can result in excessive autophagy-induced cardiac dysfunction [57]. Recently, it was reported that exercise-induced activation of AMPK leads to a reduction in oxidative stress and improvement in cardiac function in rats with diabetic cardiomyopathy [58,59]. Hence, it appears that in the present study, aerobic training probably indirectly caused the downregulation of excessive autophagy in rats with diabetic cardiomyopathy by increasing the activation of AMPK (Figure 3B). Nevertheless, it should be acknowledged that the current study is limited by the lack of measurement of ROS-related markers.

**Figure 6:**
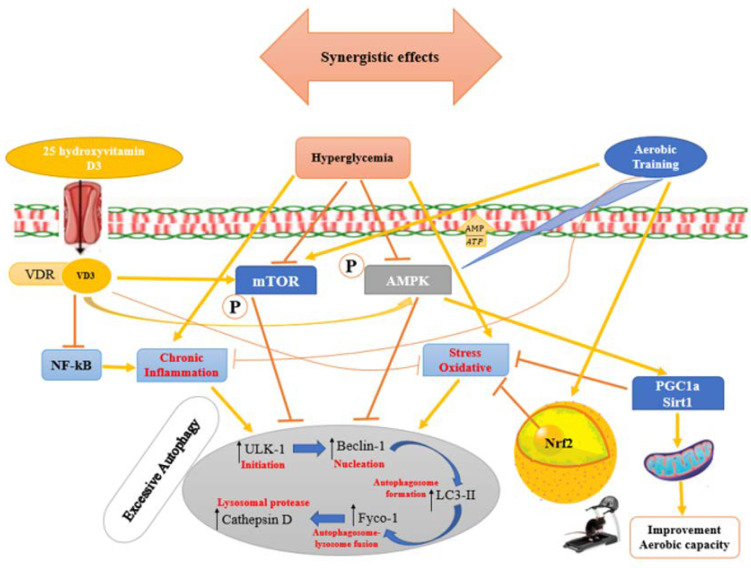
Possible schematic of signaling pathways involved in cardiac autophagy regulation in diabetic cardiomyopathy by Aerobic training and Vit D3 supplementation. Hyperglycemia inhibits autophagy-regulatory proteins such as AMPK and mTOR and can cause excessive autophagy by increasing ULK1, Beclin1, LC3II, Fyco-1 and cathepsin D proteins level directly or indirectly through chronic inflammation and oxidative stress. Aerobic training and VDR enhances AMPK and mTOR proteins levels in diabetic cardiomyopathy, thereby reducing excessive autophagy (Line arrow = Stimulatory).

Considering the greater increase in pAMPK in the DVDAT group compared to the DAT group in the present study, it appears that the interaction of aerobic training and VD3, due to synergistic impacts in improving the antioxidant capacity, probably improved the antioxidant capacity through the AMPK-Nrf-2 signaling axis. which can offer assistance diminish oxidative stress and excessive autophagy in diabetic cardiomyopathy rats [60,61]. In the present study, the autophagy markers were assessed through measurements of the protein levels of ULK-1, Beclin-1, LC3-II, Fyco-1, and Cathepsin-D. As previously mentioned, diabetic cardiomyopathy led to an increase in the protein content of markers involved in the autophagy process in the DC group compared to the CO group (Figure 6). However, aerobic training interventions and the interaction with vitamin D3 and aerobic training resulted in a significant reduction of 42% in ULK-1 levels in the DAT group and 54.4% in the DVDAT group, a significant reduction of 30.3% in Beclin-1 levels in the DAT group and 66.1% in the DVDAT group, a significant reduction of 50% in LC3-II levels in the DAT group and 67.5% in the DVDAT group, a significant reduction of 43% in Fyco-1 levels in the DAT group and 66.5% in the DVDAT group, and a significant reduction of 44% in Cathepsin-D levels in the DAT group and 58.2% in the DVDAT group compared to the DC group, which is consistent with the results of Elliott et al. [62] and Zhang et al. [63], but inconsistent with the results of Chang et al. [64], Kim et al. [36], and Pena et al. [65].

Aerobic training has been shown to improve cardiac autophagy through the mTOR-AMPK axis and prevent cardiac aging and diastolic dysfunction [58]. Autophagy-related proteins ULK-1, Beclin-1, LC3-II, Fyco-1, and Cathepsin-D had reduced levels after 8 weeks of aerobic training and its interaction with VD3, which is likely due to the inhibitory effect of mTOR and p-mTOR proteins. In the present study, 8 weeks of aerobic training led to significant increases of 61.1% and 94.2% in mTOR and pmTOR protein levels in the DAT group and significant increases of 119.4% and 125.7% in the same levels, respectively, in the DVDAT group compared to the DC group. Moreover, the mTOR and pmTOR protein level decreased by 64% and 65%, respectively, in the DC group compared to the NC group, probably due to the inhibition of the mTOR complex and phosphorylation of ULK-1 and ATG13, which initiates the formation of the double-membraned vesicles (phagophores) [66].

mTOR is one of the negative regulators of autophagy that is activated by rich nutrition and growth factors and regulates autophagy through the mTORC1 protein complex. Our findings regarding the effect of 8 weeks of aerobic training on mTOR and pmTOR levels were inconsistent with the findings of Mirsepasi et al. [67], Kwon et al. [68], Homayouni et al. [69], Liang et al. [70], and Kang et al. [71]. Among the causes of inconsistency, one is the excessive autophagy in the present study because, in this study, mTOR activity was also inhibited due to the overactivity of key autophagy initiator markers such as ULK-1, while it increased in DAT and DVDAT groups. Another reason for the inconsistency could be differences in type training intervention, animal species, or target tissue, as in this study cardiac tissue was investigated. Results from various studies have shown that training stimulates autophagy through increased activation of AMPK and inhibition of mTOR phosphorylation [39,72,73], while in the present study both pAMPK and pmTOR were significantly increased in the DAT and DVDAT groups. However, it can be said that these two factors may have independent roles in excessive autophagy retrieval at a homeostatic level in the left ventricle heart tissue of diabetic cardiomyopathy rats, although further research is needed to more thoroughly investigate the mechanisms involved in this process.

### 4.3. Effects of Vitamin D3 on Autophagy-Related Protein Levels in Rats with Diabetic Cardiomyopathy

In relation to the effect of 8 weeks of vitamin D3 on pAMPK levels, the findings indicated a significant increase of 277.7% in the DVD group and 677.7% in the DVDAT group compared to the DC group. Moreover, a significant increase of 190% and 460% in the ratio of pAMPK/AMPK ratio was observed in the DVD and DVDAT groups, respectively. Our findings regarding the effect of 8 weeks of vitamin D3 supplementation on the level of pAMPK and pAMPK/AMPK in diabetic conditions are consistent with those of Manna et al. [74], Lee et al. [75], Li et al. [76], and Herrera et al. [77], but not consistent with the findings of Choi et al. [52].

Vitamin D3 supplementation has been reported to improve glucose metabolism through the activation of autophagy signaling [39,78], which is likely mediated by AMPK activation and its related pathways. Considering the findings of the present study regarding the increased levels of pAMPK in the DVD and DVDAT groups, it appears that vitamin D3 supplementation may have played a role in regulating autophagy in diabetic cardiomyopathy rats through increasing p-AMPK level. One possible mechanism is the potential enhancement of glucose uptake and reduction of oxidative stress in the heart by increased AMPK activity [74], which may contribute to the reduction of diabetic cardiomyopathy progression [79]. However, further research is needed for a complete understanding of the mechanisms by which vitamin D3 supplementation affects the heart in this context.

The findings related to autophagy-related proteins in the DVD group showed a decrease of 19.53% in ULK-1 protein, a decrease of 42.9% in Beclin-1 protein, a decrease of 29.5% in LC3II protein, a decrease of 47% in Fyco-1 protein, and a decrease of 49.5% in Cathepsin D protein compared to the DC group. These findings are consistent with the studies by Dai et al. [80] and Guo et al. [81], but inconsistent with the studies by Li et al. [82], Mendoza et al. [83], Huang et al. [84], and Yao et al. [23]. The present study is one of the few studies that have demonstrated the excessive reduction of autophagy by vitamin D3 in diabetic cardiomyopathy rats.

There is evidence suggesting that vitamin D3 may play a role in regulating autophagy through its receptor. [85] The vitamin D receptor (VDR) is a nuclear receptor that mediates the biological effects of vitamin D [85]. Studies have shown that activation of VDR can regulate autophagy in various types of cells, including cardiomyocytes [86]. In the present study, VDR in the DVD and DVDAT groups showed an increase of 37.5% and 77.5%, respectively, compared to the DC group. It has been shown that activation of VDR leads to increased AMPK and decreased mTOR, two key regulators of autophagy [87]. Recently, it has been reported that aerobic training reduces cardiac fibrosis through increased expression of VDR due to vitamin D3 supplementation [88].

Nevertheless, it appears that the interaction of vitamin D3 and aerobic training in the present study had an additive effect on increasing VDR expression, subsequently negatively regulating autophagy excessively. In the study by Guo et al., the positive role of vitamin D receptor activation in reducing hyperactive autophagy through inhibition of FoxO1 and decreased expression of Beclin-1 and LC3II was emphasized [89], which is consistent with the findings of the present study. In the present study, vitamin D3 led to a 66.6% and 71.4% increase in mTOR and pmTOR in the DVD group, and a 119.4% and 125.7% increase in mTOR and pmTOR in the DVDAT group, respectively. The findings of the present study regarding the effect of vitamin D3 on mTOR and pmTOR are consistent with the findings of Khodir et al. [90] and Lim et al. [78].

Most studies have emphasized the role of vitamin D3 in inhibiting mTOR as a negative regulator of autophagy, which is inconsistent with the results of the present study. Notably, excessive autophagy was observed in the present study groups, and a significant reduction in the DVD group compared to the DC group was achieved through increased mTOR and p-mTOR. Since chronic inflammation is known to be a predisposing factor for excessive autophagy [91,92], and considering the anti-inflammatory role of vitamin D3 in various pathological conditions [93], it seems that in the present study, vitamin D3 may have reduced inflammation through increased activation of mTOR and decreased autophagy levels, although inflammatory markers were not investigated. Nonetheless, it can be concluded that a combination of aerobic training and vitamin D3 supplementation may have synergistic effects on reducing excessive autophagy in diabetic cardiomyopathy; however, further research is needed to achieve a comprehensive understanding of the underlying mechanisms and validation of these findings.

### 4.4. Effects of Vitamin D3, Aerobic Training, and Their Interaction on Cardiac Functional Capacity in T2DM Rats

Cardiac performance was evaluated by measuring aerobic capacity through Time to Fatigue (TTE) in rats with diabetic cardiomyopathy. The results showed a significant increase in TTE in the DAT and DVDAT groups, with this increase observed at time intervals of 2, 4, 6, and 8. However, it appears that adaptations resulting from aerobic training and its interaction with Vitamin D3 started from the initial time points and were enhanced with longer intervention periods. Various studies have investigated the role of chronic training in regulating the autophagy process [53,58,94], which may occur through different mechanisms. In the present study, the improvement in performance observed in the DT and DTVD groups can be attributed to the increased levels of AMPK induced by aerobic training and its associated pathways, including the AMPK-PGC1a-SIRT-1 pathway [95,96]. Through the stimulation of PGC1a, AMPK can activate genes involved in aerobic phosphorylation, thereby enhancing aerobic capacity in mitochondria. Additionally, it has been demonstrated that Sirt-1 can improve diabetes-related cardiomyopathy by activating AMPK and PGC1-a [97], which may also be influenced by the activation of the vitamin D receptor [98,99]. It is worth noting that the combination of aerobic training and vitamin D3 supplementation likely played a significant role in enhancing aerobic performance capacity through Sirt1 and PGC1-a-dependent signaling pathways, which are involved in regulating autophagy. 

It is important to acknowledge certain limitations in our study. Firstly, considering the association between vitamin D3 deficiency and cardiac remodeling activities, like fibrosis and apoptosis, it would be valuable for future research to address these issues by utilizing techniques such as Gomori or Mason trichrome stain and TUNEL assay. Secondly, in addition to the measured autophagy signaling proteins, our findings would be further supported by analyzing Sirt1 and PGC1-a signaling proteins.

## 5. Conclusions

In conclusion, our study suggests that aerobic training and vitamin D3 supplementation have the potential to enhance cardiac performance capacity in rats with diabetic cardiomyopathy by regulating autophagy. These findings indicate that combining these interventions may offer additional benefits for individuals with this condition. However, to validate and further understand these results, conducting clinical trials would be crucial. Such trials would provide valuable insights and help confirm the efficacy of these interventions in a clinical setting.

## Figures and Tables

**Table 1:** Aerobic training protocol during 8 weeks in T2D rats.

**Num. Week**	**MRS T1**	**First Week**	**Secound Week**	**MRS T2**	**Third Week**	**Fourth Week**	**MRS T3**	**Fifth Week**	**Sixth Week**	**MRS T4**	**Seventh Week**	**Eight Week**	**MRS T5**
**Intensity** **(% MRS T)**		55%	55%		55%	60%		60%	60%		65%	65%	
**Duration** **(min)**		30	35		40	45		50	55		60	60	

MRS T: Maximal Running Speed Test.

## Data Availability

The data presented in this study are available on request from the corresponding authors.
